# Analog Rice Based on Sago and Corn with the Addition of Moringa Leaf (*Moringa oleifera* L.) Powder as a Nutritional Vehicle for Breastfeeding Women

**DOI:** 10.3390/foods14162780

**Published:** 2025-08-10

**Authors:** Meta Mahendradatta, Tri Ela Rombe, Andi Nur Faidah Rahman, Jumriah Langkong, Abu Bakar Tawali, Dwi Ghina Nadhifa

**Affiliations:** 1 Food Science and Technology Study Program, Faculty of Agricultural Technology, Hasanuddin University, Makassar 90245, Indonesia; elatrie@gmail.com (T.E.R.); andinurfaidahrahman@unhas.ac.id (A.N.F.R.); jumriah_langkong@yahoo.com (J.L.); abubakar_tawali@unhas.ac.id (A.B.T.); dgnadhifa@gmail.com (D.G.N.); 2 Center of Excellence in Science and Technology on Food Product Diversification, Makassar 90245, Indonesia; 3 Functional Food Technology Research Group, Hasanuddin University, Makassar 90245, Indonesia; 4 Doctoral Program of Agricultural Sciences, Faculty of Agriculture, Hasanuddin University, Makassar 90245, Indonesia

**Keywords:** analog rice, breastfeeding women, corn, *Moringa oleifera*, sago

## Abstract

Breastfeeding women require specific nutrition to support the quality and secretion of breast milk, which can be achieved through the development of analog rice. Several potential alternatives to develop analog rice, including sago and corn flour, can be developed with the addition of moringa leaf powder due to its high nutritional composition and bioactive compounds, particularly high protein, iron, phytosterols, and flavonoids, which are suitable for breastfeeding women. However, as a new product, besides considering its nutritional value, developing the preferred and acceptable formulation of analog rice remains challenging. This research aims to gain the best formulation and investigate the physicochemical and sensory properties. Three formulations of analog rice were applied in this study utilizing extrusion technology, comprising sago, corn flour, and moringa leaf powder in ratio variations as follows: A, 60:37:3; B, 70:25:5; C, 80:10:10. Overall, the analog rice produced had a green color, a sticky texture, a distinct moringa aroma, and a slightly bitter taste, with Formulation C being most preferred (overall organoleptic value of 2.5, categorized as neutral), containing 6.22 ± 0.83% moisture, 1.04 ± 0.07% ash, 4.08 ± 0.17% protein, 0.46 ± 0.09% fat, 88.21 ± 0.59% carbohydrate, 3.42 ± 1.54% crude fiber, 382.62 ± 3.75 Kcal, 40.12 ± 13.38 ppm iron, 1.09 ± 0.05% sitosterol, 1.16 ± 0.03% stigmasterol, and 0.19 ± 0.07% flavonoid levels. The analog rice provides high energy and lactation-supporting bioactive compounds (iron, phytosterols, and flavonoids), demonstrating potential as a sustainable dietary intervention. This study offers a novel approach through the development of extruded analog rice, which transforms local ingredients into a functional food targeting maternal nutritional gaps by synergizing sago, corn flour, and moringa leaf powder.

## 1. Introduction

Rice is a staple food in most Asian societies and is challenging to substitute. The rising population has elevated demand for rice production and consumption [1], contributing to expanding rice import and health issues due to its high glycemic index and lack of other nutrients. Efforts have been made to enhance the functional properties of rice through cross-breeding technology, such as the riceberry developed in Thailand, with its high antioxidant content [2]. While rice breeding represents a promising strategy for enhancing the functional properties of rice, it still has some limitations, most notably its climate vulnerability and heavy reliance on agricultural land, a resource that has become increasingly scarce due to population growth and competing land-use demands [3,4]. This underscores the need for alternative strategies to mitigate the growing demand for functional foods and rising rice consumption amid limited agricultural land. One viable approach is food diversification through the development of analog rice. Analog rice mimics conventional rice and is made from a combination of various types of food ingredients, including tubers, legumes, cereals, leaves, and others [5,6]. It is prepared and consumed like rice, but offers customizable nutritional advantages for certain dietary needs, such as diabetics or nursing women.

Lactating women have higher nutritional needs than non-lactating women, as their dietary intake supports both their health and their infant’s growth through breast milk [7]. According to the data, around 30% of infants younger than 6 months old in Indonesia are not exclusively breastfed [8], and about one out of five infants aged 0–59 months were stunted [9]. This highlights the critical role of maternal nutrition in infant development. As a solution, the development of nutrient-dense analog rice can serve as a functional food tailored to the nutritional needs of nursing mothers.

Numerous studies have explored the development of functional analog rice. Estiasih et al., who developed analog rice from wild, greater, and lesser yams combined with cocoyam and arrowroot, revealed a hypotensive effect of the analog rice produced [10]. Meanwhile, Kusnandar et al. produced analog rice with a hypocholesterolemic effect made of cassava, sago, coconut dregs, and rice bran that contained γ-oryzanol as the functional compound [11]. Previous analog rice development focusing on improving the breast milk supply and quality of lactating women has also been carried out by Mahendradatta et al. using cassava, banana, katuk leaf powder, and soy lecithin in the formulation [6]. However, the utilization of sago, corn, and moringa leaf powder in producing analog rice that is beneficial for breastfeeding women has never been investigated before.

Sago (*Metroxylon sagu* Robb.), an underutilized carbohydrate-rich food source in Indonesia, contains over 90% carbohydrates, which are excellent for binding and extrusion and help maintain grain shape after cooking due to its high pasting viscosity [12,13,14]. Sweet corn (*Zea mays* L.), a nutrient-dense cereal containing essential dietary fiber, iron, minerals, and amino acids, is a promising raw material to produce functional analog rice, with its moderate amylose content (25–28%), which contributes to a firmer and less sticky texture [15,16]. Additionally, incorporating moringa leaf powder enhances nutritional value, as its bioactive compounds (phytosterol and flavonoid) act as hormone precursors to support breast milk production (lactogenic effect) [17]. Thereby, this formulation offers both the feasibility of producing analog rice and a functional food solution to meet the nutritional intake of breastfeeding women.

Furthermore, incorporating widely available local commodities into the analog rice formulation participates in supporting food product diversification programs, ensuring that staple food consumption in Indonesia does not rely solely on conventional rice. This study presents a notable advancement in developing functional food by manufacturing analog rice, which optimizes a novel composite flour formulation (sago, corn flour, and moringa leaf powder) to address nutritional gaps in maternal health. Hence, this study aims to determine the optimal formulation of analog rice produced and investigate its sensory, physical, chemical, and functional characteristics.

## 2. Materials and Methods

### 2.1. Materials

The materials used in this study were sago flour (supplied by Sagu Tani, Bogor, Indonesia), corn flour (supplied by Mugo, Bogor, Indonesia), and moringa leaf powder (supplied by Dari Bumi, Jakarta, Indonesia). All the chemicals and solvents used in this study were of analytical grade (supplied by Merck, Rahway, NJ, USA).

### 2.2. Analog Rice Production

The process of making analog rice consists of several stages, including material preparation, mixing, steaming, extrusion, molding, and drying. Sago flour, corn flour, and moringa leaf powder were prepared and weighed according to a predetermined formulation ratio, as stated in Table 1. The proportion selection was based on preliminary in-house trials and literature-reported thresholds, which represent the most viable range for scalability in terms of textural and sensorial properties. The dry ingredients were mixed separately with water for 5–10 min until evenly distributed, followed by the addition of 15% water, and then remixed for an additional 10 min. The mixture was then steamed for 30 min and extruded using a single screw extruder machine, assembled in the CV Giat Extruder Machine, Bogor, West Java, Indonesia. During the extrusion process, the mixture was shaped into rice-like grains using a cylindrical die with a 3 mm diameter and 6 mm in length, which approximates the dimensions of conventional rice kernels. The resulting extruded rice was then dried in a blower oven at 60 °C for 3 h and was stored in vacuum packaging before being analyzed.

### 2.3. Hedonic Sensory Evaluation

The sensory analysis employed in this study was the hedonic method, as prescribed by Arifin et al. [18]. Sensory analysis was conducted on 15 consumer panelists who were breastfeeding women as the intended end-consumers of the product. The hedonic scale used was a numerical scale with 3 levels of scale values: 1 = dislike, 2 = neutral, and 3 = like.

### 2.4. Evaluation of Physical Properties 

The physical characteristics of analog rice, including yield, cooking time, bulk density, water holding capacity, and swelling power, were evaluated in triplicate. The yield of analog rice was calculated by comparing its final dried weight with the initial weight of the raw materials used, as determined by the following equation:

$$Yield\left(\%\right)=\frac{Final\;weight\;of\;analog\;rice\left(g\right)}{Initial\;weight\;of\;raw\;materials\;used\;(g)}\times 100\%$$

The cooking time of analog rice was determined by observing and counting with a stopwatch from the beginning of cooking the rice using a rice cooker until the rice doneness indicator turned on, with a rice-to-water ratio of 1:1.5 *w*/*v*.

Bulk density was measured according to Chettri et al. [19] by placing the sample into the measuring cup without any compression, followed by weighing the sample. Bulk density was then calculated according to the following equation:

$$Bulk\;Density\left(\frac{g}{mL}\right)=\frac{Sample\;weight\;(g)}{Sample\;volume\;(mL)}$$

Water holding capacity (WHC) was determined by referring to Rauf and Sarbini [20]. A sample of as much as 1 g added to 10 mL of aquadest was vortexed for 2 min and left to stand for 15 min. After centrifuging for 25 min at 200 rpm, the sample was then separated from the supernatant, and the precipitate was weighed, followed by calculation of the water holding capacity using the following formula:

$$WHC\left(\%\right)=\frac{Sample\;final\;weight\;\left(g\right)}{Sample\;initial\;weight\;(g)}\times 100\%$$

The swelling power of analog rice was determined using the method described by Yudanti et al. [21]. For this, 5 g of the sample was randomly selected for the diameter measurement. The measurement was carried out using digital calipers on three side orientations: top, front, and side. It was carried out on the rice both before and after immersion. The measurement of the diameter of analog rice grain was calculated as follows:

$$\mathrm{Analog}\;\mathrm{rice}\;\mathrm{diameter}\;\mathrm{measurement}\;(\text{Ø})\;=\frac{Ø1+Ø2+Ø3}{3}$$

The swelling power of analog rice grains was determined with a formula:

$$\mathrm{Swelling}\;\mathrm{power}\;(\%)\;=\frac{analog\;rice\;diameter\;after\;immersion\;\left(mm\right)-before\;immersion\;(mm)}{analog\;rice\;diameter\;before\;immersion\;(mm)}\times 100\%$$

### 2.5. Chemical Properties Evaluation

#### 2.5.1. Proximate Contents

Proximate contents, consisting of moisture, ash, protein, fat, and crude fiber levels, were analyzed according to the Association of Official Analytical Chemists [22], and the total calories of analog rice were determined according to the Atwater system [23], which estimates the energy content of food by the gross energy content of dietary proteins, fats, and carbohydrates, calculated as follows:

Total Calories = (4.1 × Protein Content) + (9.3 × Fat Content) + (4.1 × Carbohydrate content)

#### 2.5.2. Iron Analysis

The iron content of analog rice was determined using the method described by Krismaputri et al. The 5 g sample was placed in the exchange rate, then heat-treated in a furnace for 6 h at 600 °C. Before being diluted with distilled water, the ash content was weighed, combined with 25 mL of HCl, and heated for 30 min. After transferring a 5 mL aliquot of the ashing solution to a 25 mL flask and adjusting the pH to 3.5 ± 1 with two drops of sodium acetate and bromophenol blue, 4 mL of 1,10-phenanthroline was added. After dilution and shaking, the solution was left for 1 h. The solution was shaken and diluted before being left for an hour. UV–vis spectroscopy was used to measure absorbance at 515 nm, and a standard calibration curve was used to calculate the iron content [24].

#### 2.5.3. Sitosterol and Stigmasterol Analyses

Sitosterol and stigmasterol levels of analog rice were measured by referring to Indrayanto et al. [25], in which samples weighing up to ±0.25 g were placed in a 25 mL volumetric flask. Alcohol was then added, along with roughly 1/3 of a volumetric flask, shaken for two hours, and filtered. After spotting 5 µL of the filtrate on the TLC plate, 5 µL of sitosterol and stigmasterol standards containing 100 ppm were added. After about 45 min of operation with the eluent CHCl_3_: ethanol: ethyl acetate, we measured the sitosterol and stigmasterol using a TLC scanner set to 285 nm and 264 nm, respectively.

#### 2.5.4. Flavonoid Analysis

The flavonoid content was determined following Chang et al. [26] using a quercetin standard curve. A stock solution (1000 ppm) was prepared by dissolving 25 mg of quercetin standard in 25 mL of 96% ethanol. Then, 1 mL of the stock solution was pipetted, and the volume was made up to 10 mL. As much as 5 mL was pipetted again, and the volume was made up to 50 mL. A standard 100 ppm quercetin solution was made into several concentrations (2–10 ppm). Both standards and samples (25 mg in 25 mL ethanol, diluted 1:10) were mixed with 3 mL ethanol, 0.2 mL AlCl_3_, 0.2 mL 1 M potassium acetate, and 5.6 mL aquabidest. After incubation for 30 min at room temperature, absorbance was measured using a UV–visible spectrophotometer at a wavelength of 440 nm, with triplicate measurements per sample.

### 2.6. Statistical Analysis

The data were processed using a completely randomized design (CRD) with three replications using Microsoft Excel 2010. Data obtained for each sensory test parameter and physical and chemical analysis were analyzed by analysis of variance (ANOVA) with three replications. The differences for each treatment were further tested using Duncan’s test using SPSS 24. Principal Component Analysis (PCA) to explore the relationship between the analog rice formulations and their physical and chemical properties was performed using Jupyter Notebook version 7.4.4.

## 3. Results and Discussion

### 3.1. Hedonic Sensory Evaluation

**Figure 1:**
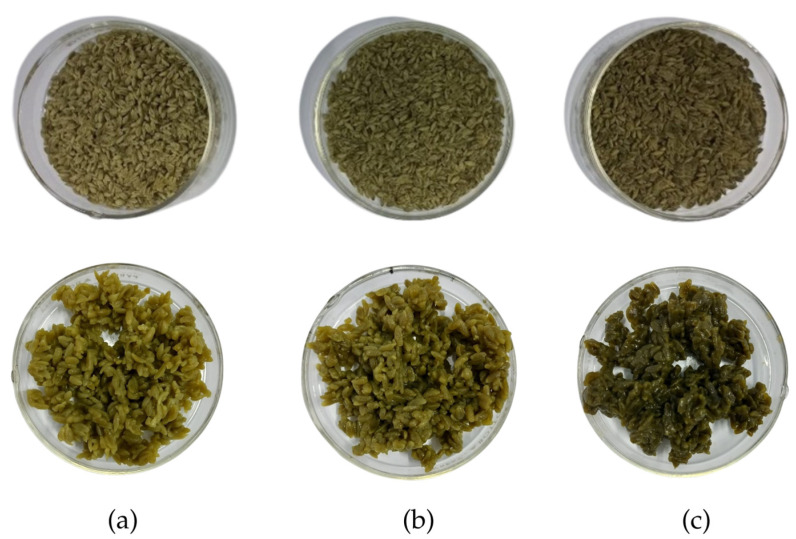
Raw and cooked analog rice formulations of sago flour, corn flour, and moringa leaf powder. (**a**) Formulation A (60:37:3%), (**b**) Formulation B (70:25:5%), and (**c**) Formulation C (80:10:10%).

One of the most common consumer sensory evaluation techniques is a hedonic method, which measures the degree of preference or liking of a food product, based on a structured scale. This study employed a three-point scale of hedonic sensory evaluation, designed to provide unambiguous ‘accept’ or ‘reject’ data for formulation refinement from the targeted consumers, who were breastfeeding mothers prone to fatigue during the postpartum period. The raw and cooked analog rice produced can be seen in Figure 1. In the color, aroma, texture, and overall acceptance parameters, analog rice Formulations A and B showed insignificant differences (*p* > 0.05) but were significantly different (*p* < 0.05) from Formulation C, whereas in the taste parameter, all three formulations were significantly different (Table 2). The color of the analog rice developed was light to dark green, originating from the moringa leaf powder’s color, with color intensity increasing proportionally with its concentration. Sensory evaluation revealed that all formulations were rated as “neutral” in preference, though panelists favored samples with lower green intensity. While moringa chlorophyll serves as a natural green pigment for food applications, higher concentrations reduce consumer acceptability [27].

The raw materials used influenced the aroma of the analog rice produced. The greater the amount of moringa leaf powder added, the less preferred the aroma of analog rice. This is due to the green or herbal aroma of the moringa leaf powder, which intensifies as the proportion increases, resulting in an unpleasant odor to consumers [28,29]. Although the texture preference values of all three formulations were included in the “neutral” category, with a slightly fluffy and sticky texture, Formulation C, which had the highest sago and moringa leaf powder ratio, was more unfavorable. The stickiness of the analog rice increased along with the rising ratio of sago flour, as its high amylopectin content plays a key role in increasing viscosity [30], giving consumers a less favorable chewing experience of rice. Formulation A (60% sago flour, 37% corn flour, 3% moringa leaf powder) showed optimal taste acceptability (score: 2.49, ‘neutral to like’), while Formulation C (80% sago flour, 10% corn flour, 10% moringa leaf powder), with higher moringa content, received lower preference. The inherent bitterness of moringa strengthens the perceived bitterness in the product as the concentration increases [31]. Overall, the acceptance rates of Formulations A and B were insignificantly different, with the former holding the highest value. In contrast, Formulation C was the least favorable, presumably because of the higher concentration of moringa leaf powder added.

### 3.2. Physical Properties

The results of the physical properties of analog rice, including yield, cooking time, bulk density, water holding capacity, and swelling power, are shown in Table 3. Yield is defined as the dry product to raw material weight ratio and is essential for calculating the processing’s influence on the final product [32]. This enables precise determination of process optimization, a critical factor in determining the feasibility of future commercialization. The yield exhibited no significant differences among the formulations (60.50% to 63%). Material loss happened during the extrusion process, primarily due to dough residue formation in the mold. This occurs when the raw materials reach their gelatinization temperature during heating, causing adhesion and hardening of unextruded portions [33,34].

The cooking time (13.85–14.26 min), bulk density (0.60–0.63 g/mL), water holding capacity (73.21–75.08%), and swelling power (25.01–29.10%) of analog rice formulations demonstrated no significant variations. Analog rice cooking time is a parameter used to determine how long it takes to gelatinize completely, which is influenced by the raw materials’ composition and processing [35]. Analog rice’s cooking time was shorter than conventional rice due to the formation of cavities during drying, enhancing water absorption [36]. Bulk density, a parameter to determine the space needed to pack the product, showed an increasing value with higher sago and moringa content, indicating a denser structure with fewer voids [37]. The water holding capacity and swelling power are governed by starch composition, where amylopectin’s water-reactive branches enhance both water absorption and swelling capacity [38], while amylose’s strong intermolecular bonds limit expansion [39]. Inherent problems with extruding composite flours are indicated by the relatively high observed standard deviations in the yield and swelling power of analog rice. Therefore, to reduce variability, future research could employ more replicates (n ≥ 5) or sophisticated process controls.

### 3.3. Chemical Properties

#### 3.3.1. Proximate Content

The proximate contents of analog rice formulations, as stated in Table 4, exhibited significant differences for ash, protein, and fat levels (*p* < 0.05). In contrast, moisture, carbohydrate, and fiber levels, as well as total calories, had insignificant differences (*p* > 0.05). The moisture content of the analog rice ranged from 6.22% to 7.06%, which is within the acceptable limit set by the Indonesian National Standard for rice, defined as less than 14% [40]. Measuring moisture content in food products is crucial for determining their shelf life, safety, and quality [41].

The ash level of the analog rice decreased with lower corn flour proportions in the formulation, all meeting the requirement of ≤3.50% for Mother’s Milk Complementary Food’s ash content set by the Indonesian National Standard [42]. The higher ash content in Formulation A resulted from corn flour’s rich mineral composition, including Fe (23.74 mg/100 g), Ca (557.25 mg/100 g), K (1133.50 mg/100 g), Mg (621.25 mg/100 g), and P (1389.3 mg/100 g), among other essential minerals [43].

While Formulation A of analog rice had the highest protein content of 4.08%, incorporating the most corn flour, Formulation C had the second highest protein content at 3.73%, including the most moringa leaf powder. Corn flour has a protein content of about 80%, whereas moringa leaves contain about 60% protein, contributing to the analog rice’s protein content. Protein plays a crucial role in maintaining the breast milk supply of breastfeeding mothers and building the milk protein to support sufficient nutrition for the infant’s growth [44,45].

The fat level of the analog rice produced was in a slightly small amount, around 0.27–0.62%, reflecting the lean composition of its primary ingredients. Sago flour is predominantly composed of carbohydrates, containing only a small amount of fat (0.17–0.21%) [46]. Similarly, corn flour is also a carbohydrate source, having a relatively low fat content of 3.9% [47]. Although moringa leaf powder has a higher fat content (4.03–9.51%) [48], its small amount in the formulation resulted in negligible lipid contribution to the final product.

The carbohydrate levels of all three formulations of analog rice were similar, at about 88%. Since developing analog rice is expected to be able to replace regular rice, the primary ingredient used in the formulations should be rich in carbohydrates, which, in this study, was derived from sago flour and corn flour. Sago flour has a carbohydrate content of around 90% [49], while corn flour contains a lower total carbohydrate content than sago, of 83.83% [50], making them suitable as carbohydrate sources in analog rice to provide energy for nursing mothers.

An indigestible component of plant-based diets, crude fiber has been widely known to play a vital role in supporting digestive health [51,52]. The analog rice’s crude fiber levels showed similar results, around 2.41–3.42%. Corn flour’s crude fiber varies from 2.41% to 9.03% [53,54], contributing to a higher crude fiber level in the analog rice with a higher corn concentration added.

Total calories refer to the total amount of energy derived from food’s macronutrients (carbohydrates, proteins, fats). All three analog rice formulations’ total calories exhibited insignificant differences of around 380–382 kcal, higher than that of regular rice, around 309.62–342.88 kcal/100 g [55]. The Ministry of Health of the Republic of Indonesia recommends a higher calorie intake for lactating women, 2580 kcal/day, than for pregnant women, 2550 kcal/day [56]. Therefore, the analog rice with high calories produced in this study is considered a promising alternative food product to support the higher calorie intake of lactating women.

#### 3.3.2. Iron Content

**Figure 2:**
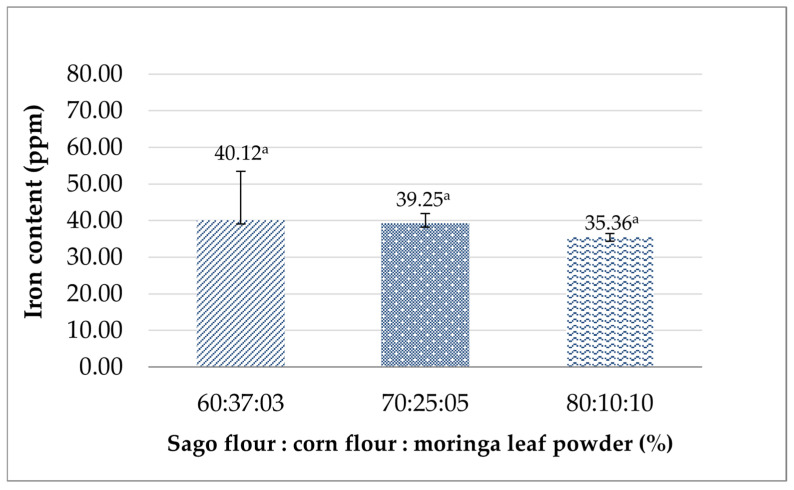
The effects of the sago flour, corn flour, and moringa powder proportions on the iron content of analog rice. Similar superscripts denote insignificant differences (*p* > 0.05).

Iron is the most abundant micro-mineral found in the adult human body, accounting for around 3–4 g needed to form hemoglobin (Hb) [57]. Breastfeeding women need a higher iron intake than non-breastfeeding women, since it is required in order to form red blood cells to prevent anemia, which lactating women are prone to, and maintain the iron quality in breast milk for the infant [58]. The result (Figure 2) showed that the iron content of the analog rice produced ranged from 35.36 to 40.12 ppm and was not significantly different (*p* > 0.05). As the ratio of moringa leaf powder to analog rice formulation increases, the lower the iron content of the analog rice obtained. This finding is inconsistent with previous studies, which found that moringa is considered one of the lactation-inducing products capable of initiating, maintaining, and/or increasing the breast milk supply of nursing mothers [59,60]. Rissa also found that moringa capsule supplementation was effective in increasing Hb levels in pregnant women [61]. The contrary results found in this study may be attributed to several factors, including processing, the storage environment, and sample preparation prior to testing. Iron’s stability in food products is prone to decrease due to its pro-oxidant characteristic and easy reactivity with other food components [62,63]. This is supported by Lubaale et al.’s finding, which revealed that the extrusion process of sorghum-based food decreased the bioaccessible iron content and the percentage of bioaccessible iron [64]. Additionally, low shear and hot extrusion can retain iron content better than high shear and cold extrusion due to the A-type starch polymorphism in cold-extruded products [65,66].

#### 3.3.3. Sitosterol and Stigmasterol Content

**Figure 3:**
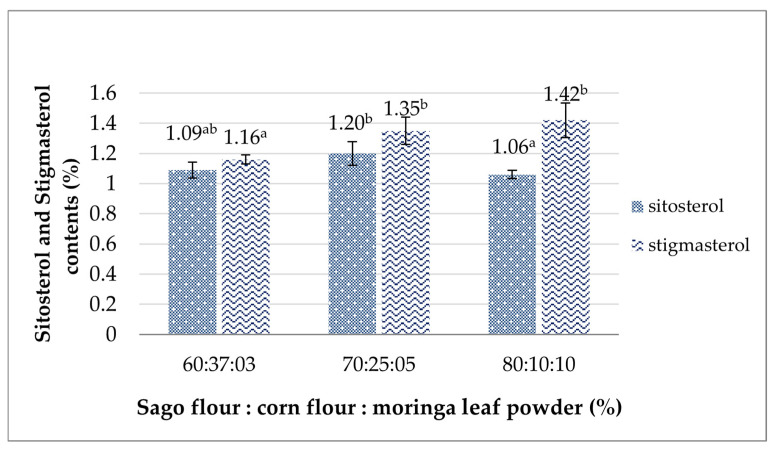
The effects of the sago flour, corn flour, and moringa powder proportions on the analog rice’s sitosterol and stigmasterol contents. Different superscripts denote significant differences (*p* < 0.05).

The phytosterol contents analyzed in this study include sitosterol and stigmasterol, both of which are bioactive compounds, and showed a significant difference (*p* > 0.05) presented in Figure 3. The results obtained in the analog rice with ratios of sago flour, yellow corn flour, and moringa leaf flour of 60%:37%:3%, 70%:25%:5%, and 80%:10%:10%, respectively, were 1.09%, 1.20%, and 1.06% for sitosterol content, whereas the stigmasterol content was 1.16%, 1.35%, and 1.42%. The second formulation with 5% moringa leaf powder exhibited a higher sitosterol level, while that with 10% moringa leaf powder had a greater stigmasterol level. Asrifah et al. found that white stalk moringa leaf flour contained 616.13 ppm sitosterol and 6.80 stigmasterol [67]. In comparison, Talreja and Goswami revealed sitosterol and stigmasterol in the leaflets of moringa to be as much as 9.37 mg/100 g and 5.33 mg/100 g, respectively [68]. These phytosterols derived from moringa leaves have been proven to have a lactogenic effect, a milk-increasing effect, by producing more estrogen, which supports the stimulation of gland duct proliferation for milk production [69]. Existing research has reported an increase in the amount of breast milk in the group of breastfeeding mothers given moringa leaf capsules up to 47% [70] and 66.2% for nursing mothers given moringa leaf powder [71]. Therefore, integrating moringa leaf powder into food product formulations, like the analog rice, presents a viable strategy to address the nutritional needs of lactating mothers.

#### 3.3.4. Flavonoid Levels

**Figure 4:**
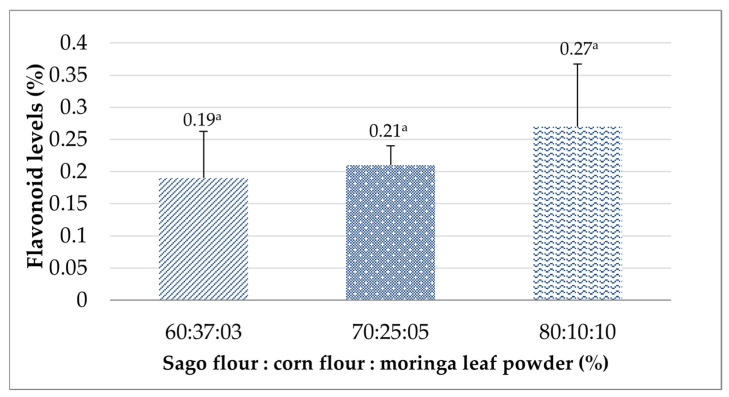
The effects of the sago flour, corn flour, and moringa powder proportions on the flavonoid levels of the analog rice. Different superscripts denote significant differences (*p* < 0.05).

Flavonoids are secondary metabolites abundantly found in plants. They have potential functions, such as antioxidant, anticancer, antimicrobial, antiviral, and antibiotic functions, among other biological activities beneficial for health [72]. The flavonoid results found in this study demonstrated a non-significant effect for all formulations (*p* > 0.05), with values ranging from 0.19% to 0.27% (Figure 4). An 80:10:10 ratio of sago flour, corn flour, and moringa leaf powder possessed the highest flavonoid level, likely attributable to the greater proportion of moringa leaf powder in the formulation. Moringa leaves have been widely known for their health-promoting effects, which earlier studies found to be associated with their higher amount of flavonoids than in other vegetables and fruits [73]. Total flavonoid contained in moringa leaf powder is 1.41 g/100 g [74]. The most prevalent type of flavonoids found in moringa leaves are quercetin, kaempferol, apigenin, luteolin, and myricetin [75]. Flavonoids hold a significant role in facilitating and accelerating breast milk production by stimulating the breastfeeding hormones prolactin and oxytocin. Pujiastuti et al. revealed that breastfeeding mothers who consumed moringa leaf cookies up to five pieces for 14 days could effectively improve the babies’ weight compared with the control group [76]. A similar result was also reported by Pratiwi and Srimiati, who gave moringa leaf pudding to nursing women and found that there was a significant increase in the babies’ weight after the intervention for 7 days [77].

### 3.4. Principal Component Analysis (PCA) of Physical and Chemical Characteristics of Analog Rice Formulations

**Figure 5:**
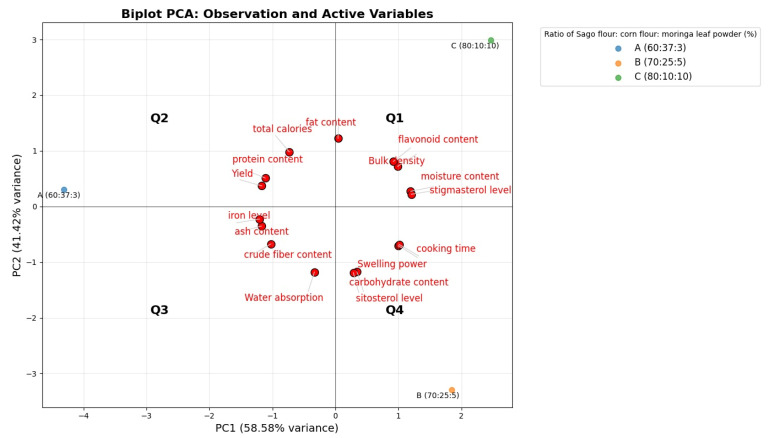
A biplot graph to clarify the relationships among the three formulations of analog rice according to their physical and chemical characteristics.

Based on the Principal Component Analysis (PCA), a biplot graph was obtained to explore the relationships between the physical and chemical characteristics of analog rice in each formulation consisting of different ratios of sago, corn flour, and moringa leaf powder. Strong correlations are indicated by angles less than 90°, whereas no correlation is indicated by angles greater than 90°. The groups of analog rice formulations were divided into three distinct clusters on the basis of the analysis shown in Figure 5. Formulation A, located in Quadrant two and adjacent to Quadrant three, shows proximity with parameters such as yield, total calories, protein, iron, and ash contents, which is attributed to its composition, as it contains a higher amount of corn flour and less sago, resulting in high levels of these parameters. Formulation B, which comprises 70% sago, 25% corn flour, and 5% moringa leaf powder, is clustered with parameters including swelling power, cooking time, carbohydrate content, and sitosterol levels. Meanwhile, parameters such as flavonoid content, stigmasterol levels, moisture content, and bulk density exhibit positive correlations and are grouped within the same cluster as Formulation C (80% sago, 10% corn flour, and 10% moringa leaf powder). The analysis indicates that higher concentration of sago (70–80%) correlates with longer cooking times, high swelling power, moisture content, and bulk density, associated with its starch components. In addition, the proportion of moringa flour (5–10%) is linked to its proximity with sitosterol, stigmasterol, and flavonoid levels, which are bioactive compounds which offer nutritional advantages for breastfeeding women [78].

## 4. Conclusions

Analog rice made from sago and corn flour, with added moringa leaf powder, offers a promising substitute for conventional rice, as well as a functional food in supporting the breast milk secretion of nursing mothers. The analog rice formulation comprising 60% sago, 37% corn flour, and 3% moringa leaf powder served as the best formulation, based on the panelists’ preference, having a light green hue, a fluffy and less sticky texture, a slight moringa aroma, and a neutral, less bitter taste. However, while the lesser amount of moringa leaf powder in the analog rice formulation was more favorable for its sensory properties, it exhibited small levels of sitosterol and flavonoids, the key compounds of lactating-inducing precursors. Hence, exploring the development of its sensory characteristics is still needed to make it fully accepted while optimizing its functional compounds. In addition, a clinical trial can be conducted to investigate the efficacy of this formulation. Future research is encouraged to examine cost–benefit and nutritional comparisons between traditional fortified staples and analog rice, thereby better positioning this innovation within maternal nutrition interventions.

## 5. Patents

This work is registered under Indonesian Patent No. P00202300259 by the Directorate General of Intellectual Property, Ministry of Law and Human Rights, Republic of Indonesia (Issued 3 August 2023).

## Figures and Tables

**Table 1:** Analog rice formulation of sago flour, corn flour, and moringa leaf powder at different ratios.

Sample Code	Sago Flour (%)	Corn Flour (%)	Moringa Leaf Powder (%)	Total (%)
A	60	37	3	100
B	70	25	5	100
C	80	10	10	100

**Table 2:** Hedonic sensory evaluation of analog rice. A, B, and C represent the sample codes provided in Table 1.

Analog Rice Formulation	Color	Aroma	Texture	Taste	Overall Acceptance
A	2.51 ± 0.05 ^b^	2.53 ± 0.02 ^b^	2.48 ± 0.07 ^b^	2.49 ± 0.06 ^c^	2.50 ± 0.02 ^b^
B	2.47 ± 0.10 ^b^	2.43 ± 0.06 ^b^	2.48 ± 0.08 ^b^	2.31 ± 0.06 ^b^	2.42 ± 0.08 ^b^
C	2.07 ± 0.09 ^a^	2.12 ± 0.07 ^a^	2.17 ± 0.05 ^a^	1.91 ± 0.08 ^a^	2.07 ± 0.11 ^a^

Data are expressed as means ± SD. Different superscripts within a column denote significant differences (*p* < 0.05).

**Table 3:** Physical properties of analog rice.

% Sago Flour: Corn Flour: Moringa Leaf Powder	Yield (%)	Cooking Time (Minutes)	Bulk Density (g/mL)	Water Holding Capacity (%)	Swelling Power (%)
A (60:37:3)	63.00 ± 12.60 ^a^	13.85 ± 0.30 ^a^	0.60 ± 0.01 ^a^	74.46 ± 0.98 ^a^	25.01 ± 3.01 ^a^
B (70:25:5)	60.50 ± 16.89 ^a^	14.26 ± 0.17 ^a^	0.61 ± 0.01 ^ab^	75.08 ± 1.46 ^a^	29.10 ± 8.03 ^a^
C (80:10:10)	61.08 ± 8.18 ^a^	14.06 ± 0.07 ^a^	0.63 ± 0.01 ^b^	73.21 ± 1.36 ^a^	27.03 ± 1.74 ^a^

Data are expressed as the mean ± SD. Different superscripts within a column denote significant differences (*p* < 0.05).

**Table 4:** Proximate content and total calories of analog rice.

% Sago Flour: Corn Flour: Moringa Leaf Powder	Moisture Level (%)	Ash Level (%)	Protein Level (%)	Fat Level (%)	Carbohydrate Level (%)	Crude Fiber Level (%)	Total Calories (Kcal)
A (60:37:3)	6.22 ± 0.83 ^a^	1.04 ± 0.07 ^c^	4.08 ± 0.17 ^b^	0.46 ± 0.09 ^b^	88.21 ± 0.59 ^a^	3.42 ± 1.54 ^a^	382.62 ± 3.75 ^a^
B (70:25:5)	6.80 ± 0.43 ^a^	0.70 ± 0.02 ^b^	3.53 ± 0.31 ^a^	0.27 ± 0.02 ^a^	88.71 ± 0.74 ^a^	3.04 ± 0.28 ^a^	380.67 ± 1.60 ^a^
C (80:10:10)	7.06 ± 0.34 ^a^	0.50 ± 0.13 ^a^	3.72 ± 0.13 ^ab^	0.62 ± 0.02 ^c^	88.11 ± 0.51 ^a^	2.41 ± 0.51 ^a^	382.21 ± 1.56 ^a^

Data are expressed as the mean ± SD. Different superscripts within a column denote significant differences (*p* < 0.05).

## Data Availability

The data supporting this study are available within the article. Additional inquiries may be directed to the corresponding author.
